# Rotator cuff tear patterns: MRI appearance and its surgical relevance

**DOI:** 10.1186/s13244-024-01607-w

**Published:** 2024-02-27

**Authors:** Alexeys Perez Yubran, Luis Cerezal Pesquera, Eva Llopis San Juan, Fernando Idoate Saralegui, Alvaro Cerezal Canga, Antonio Cruz Camara, Gustavo Matheus Valdivieso, Carolina Pisanti Lopez

**Affiliations:** 1 Department of Radiology, IBERORAD, Carrer Valencia 226, Principal, primera, Barcelona, 08007 Spain; 2 Department of Radiology, Diagnóstico Médico Cantabria, Santander, Spain; 3 Department of Radiology, Hospital de La Ribera, Valencia, Spain; 4 Department of Radiology, Mutua Navarra, Pamplona, Spain; 5 Department of Orthopaedic Surgery and Traumatology, Hospital Universitario La Paz, Madrid, Spain; 6 Department of Arthroscopic Surgery, Hospital Santa Clotilde, Santander, Spain; 7 Department of Shoulder Surgery, Hospital Ortopedico Infantil, Caracas, Venezuela

**Keywords:** Rotator cuff, Delaminated, Massive tears, Surgery, Outcome

## Abstract

A new perspective on rotator cuff anatomy has allowed a better understanding of the patterns of the different rotator cuff tears. It is essential for radiologists to be aware of these different patterns of tears and to understand how they might influence treatment and surgical approach. Our objective is to review the arthroscopy correlated magnetic resonance imaging appearance of the different types of rotator cuff tears based on current anatomical concepts.

**Critical relevance statement** Knowledge of the characteristics of rotator cuff tears improves our communication with the surgeon and can also make it easier for the radiologist to prepare a report that guides therapeutic conduct and serves as a prognosis for the patient.

**Key points**

• There is no universally accepted classification for RC tears.

• New patterns such as delamination or myotendinous junction tears have been defined.

• The most difficult feature to assess in full thickness tears on MRI is the pattern.

• Fatty infiltration of the RC tendons is crucial in the prognosis and outcome.

• The radiological report is an effective way of communication with the surgeon.

**Graphical Abstract:**

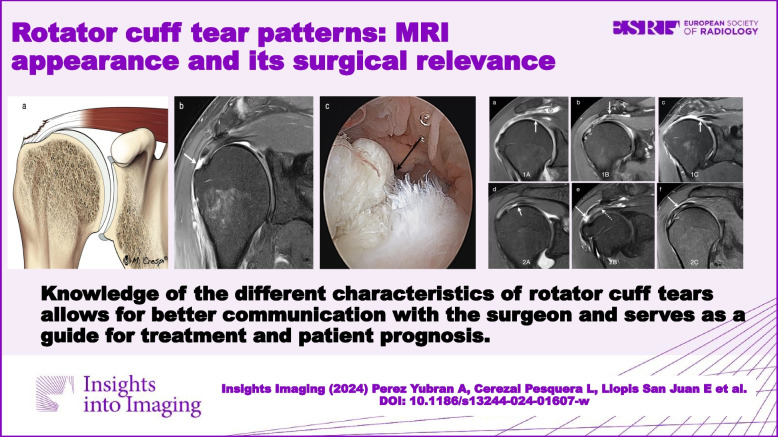

## Introduction

Chronic degenerative processes or traumatic injuries of the rotator cuff are the most common tendon injury related problem in adult population. RC injuries might cause a debilitating impact *on* the fragile dynamic instability of the shoulder with pain and functional impairment in daily routine activity.

Advances in anatomy, imaging, and arthroscopy have provided opportunities for improved treatment strategies, based on a better understanding of the different patterns of RC tears.

Previous divergent perspectives on and complexity of RC disease have resulted in different types of classification systems [1], most of which are not standardized or universally accepted. The international Society of Arthroscopy, Knee Surgery and Orthopedic Sports Medicine (ISAKOS) group published an expert consensus manuscript trying to establish a guideline to predict patients’ outcome and to assist in therapeutic decision making [2] (Table 1). However, new tear patterns have recently been identified which do not fit in the ISAKOS or classic classifications [3, 4]. These new patterns can be explained by anatomical features that are visualized with MRI. MRI thus remains the gold standard imaging method for diagnosing cuff tears, for depicting features bearing prognostic implications, allowing therapeutic decision-making as well as planning surgery for those potential candidates for surgical reconstruction [2].

**Table 1:** ISAKOS rotator cuff classification system for rotator cuff tears

Location (L)	Extension (E)	Pattern (P)	Fatty atrophy (A)	Retraction (R)
Partial thickness posterosuperior	< 50% thickness > 50% thickness	Articular Bursal Interstitial	SS0 IS0 SS1 IS1 SS2 IS2 SS3 IS3 SS4 IS4	
Full thickness posterosuperior (SS-IS)	C1 C2 C3 C4 (Massive)	C, U, L, rL (reverse L)		1,2,3
Anterior (SSC)	1,2,3,4,5		SSC 0 SSC 1 SSC 2 SSC 3 SSC 4	

Reprinted from ISAKOS Shoulder Concepts 2013: consensus and concerns By G. Arce et al. (eds.) Springer. New york.2013

*SS* supraspinatus, *IS* infraspinatus, *SSC* subscapularis

Geometric Classification system of rotator cuff tears: *C* crescent-shaped, *L* L-shaped, *rL *reverse-shaped, *U* U-shaped [5]

The goal of this paper is by providing a comprehensive anatomical and radiological description of the RC tear patterns in MRI. We will also briefly review the most common surgical techniques.

## Key anatomical concepts

The anatomy of the RC is complex. It is well known that the insertions of the RC are not based on one single insertion, but they have main and secondary insertions where different tendons blend [6, 7]. RC tendons can be divided into an anterior tendon, the subscapularis, and posterior tendons, the supraspinatus, infraspinatus and teres minor.

**Fig. 1:**
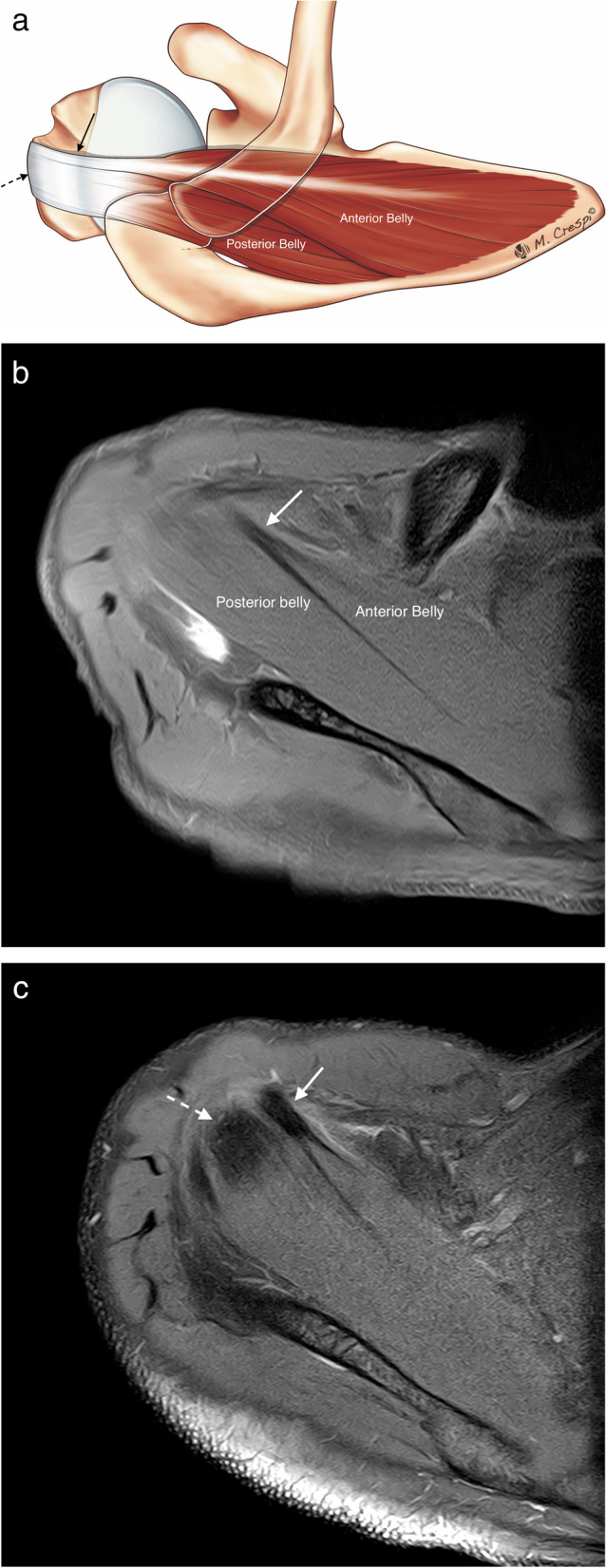
Supraspinatus components. **a** Drawing showing the bellies and tendons of supraspinatus. **b**, **c** Axial fat-suppressed PD-weighted MRI showing the anterior belly with its long tendon and insertion (arrows) and the posterior belly with a broader tendon (dashed arrows)

The supraspinatus (SS) originates from the posterior aspect of the scapula in the SS fossa above the spine of the scapula and inserts in horizontal and anterior directions onto the greater tuberosity of the humeral head. The SS is divided into two components (anterior and posterior) and each of them is subdivided into three *parts* (superficial, middle, and deep) [3, 8]. The anterior belly of the SS is voluminous and tubular, has a bipennate configuration, and generates greater contractile forces than the posterior belly, partly because of its long intramuscular tendon [3, 9] (Fig. 1). In contrast, the posterior belly muscle is smaller, is unipennate, and its tendon is flatter and wider. The posterior belly generates less contractile forces. Despite the anterior muscle belly being almost double the size of the posterior belly, the ratio for the tendon cross-sectional area between anterior and posterior is 0,9:1. The difference in contractile load together with this discrepancy in tendon size, increases the stress in the anterior belly, making it especially susceptible to myotendinous injuries and tendon injuries [3].

The infraspinatus (IS) is the second RC muscle in size. It arises from the dorsal surface of the infraspinatus fossa of the scapula and inserts onto the greater tuberosity of the humerus, forming a functional unit with the SS tendon. Its fibers run parallel to the teres minor but are separated by a thick fascia.

**Fig. 2:**
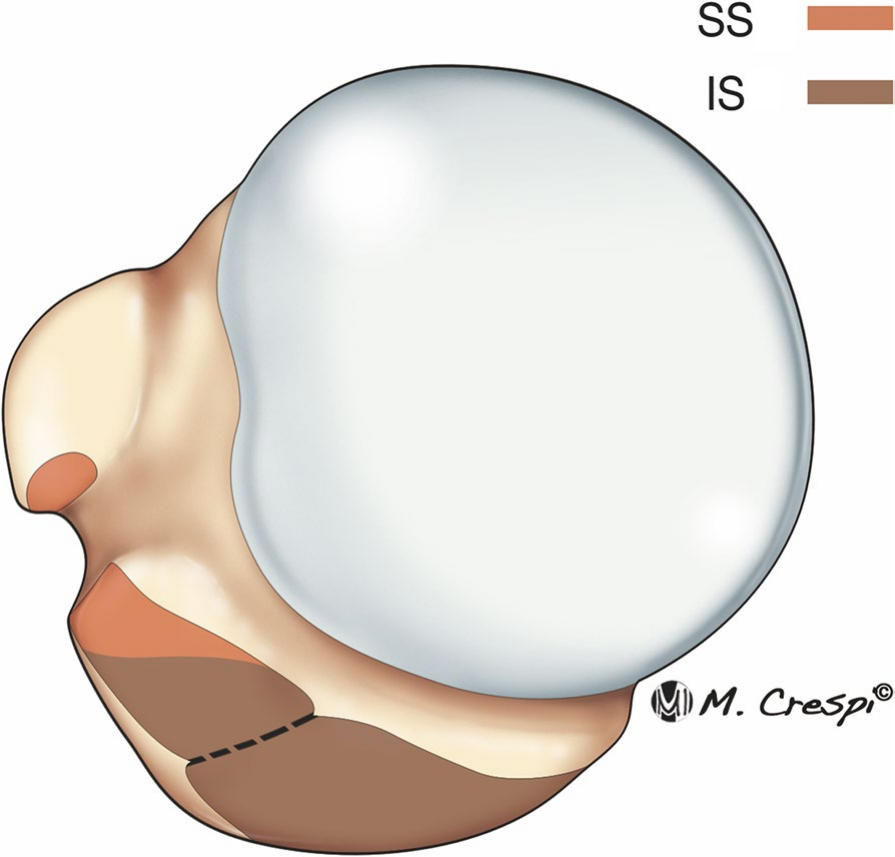
Normal supraspinatus and infraspinatus insertions. The SS inserts on a small triangular area in the humeral head, extending from the bicipital groove anteromedially to the top of the greater tuberosity. The IS footprint instead has a broader insertion and covers the posterior border of the SS superiorly and inserts onto the anterolateral area and the middle facet of the greater tuberosity

The main osseous insertions of the SS and IS cuff tendons form a footprint that is a three-dimensional area on the humeral head. The SS insertion has a triangular shape extending from the bicipital groove anteromedially to the top of the greater tuberosity. The IS insertion has a trapezoid shape, is wider, covers the posterior border of the SS superiorly and inserts onto the anterolateral area and the middle facet of the greater tuberosity [10–12] (Fig. 2). The insertion on this shared footprint suggests that the IS may be more frequently affected in RC tears than we usually thought and could be one of the reasons explaining the high incidence of fatty atrophy of the IS occurring in isolated tears of the SS. The footprint insertions are important in surgical planning to adequately restore shoulder function [12–14].

**Fig. 3:**
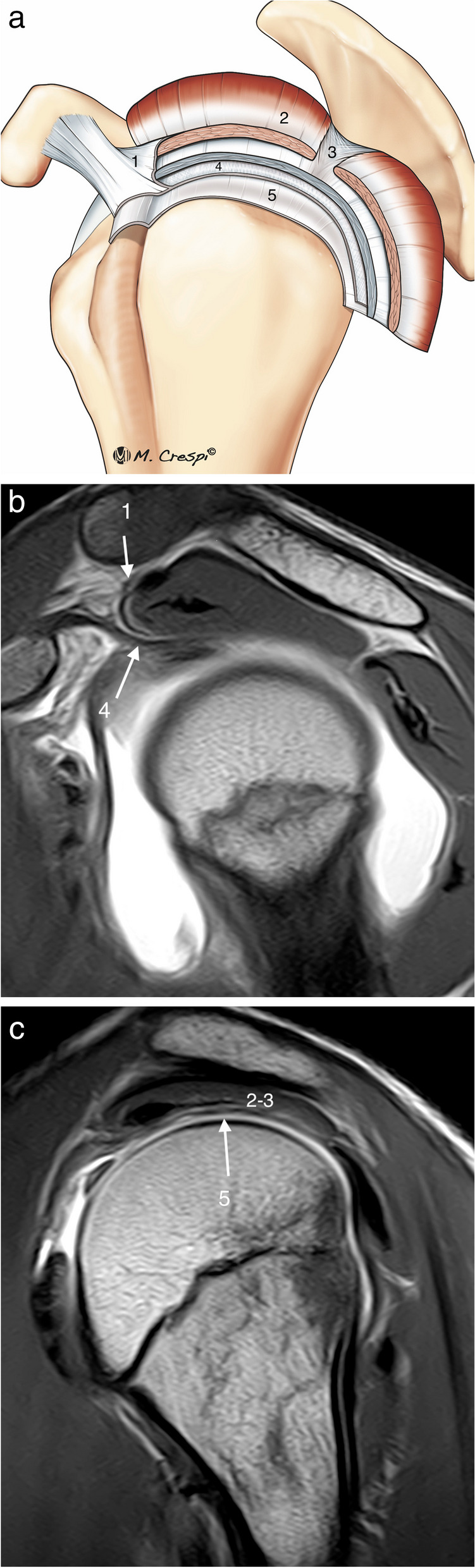
Normal supraspinatus and infraspinatus ultrastructure. **a** Drawing shows the different layers of RC. **b, c** Oblique-sagittal PD-weighted MRI arthrography. Layer 1, superficial fibers of the coracohumeral ligament; layer 2 are dense parallel collagen fibers from SS and IS tendons or bursal fibers and layer 3 are smaller collagen fibers of less uniform orientation or articular fibers. These are very difficult to differentiate even with high field MRI. Layer 4 is the rotator cable and layer 5, superior capsule

**Fig. 4:**
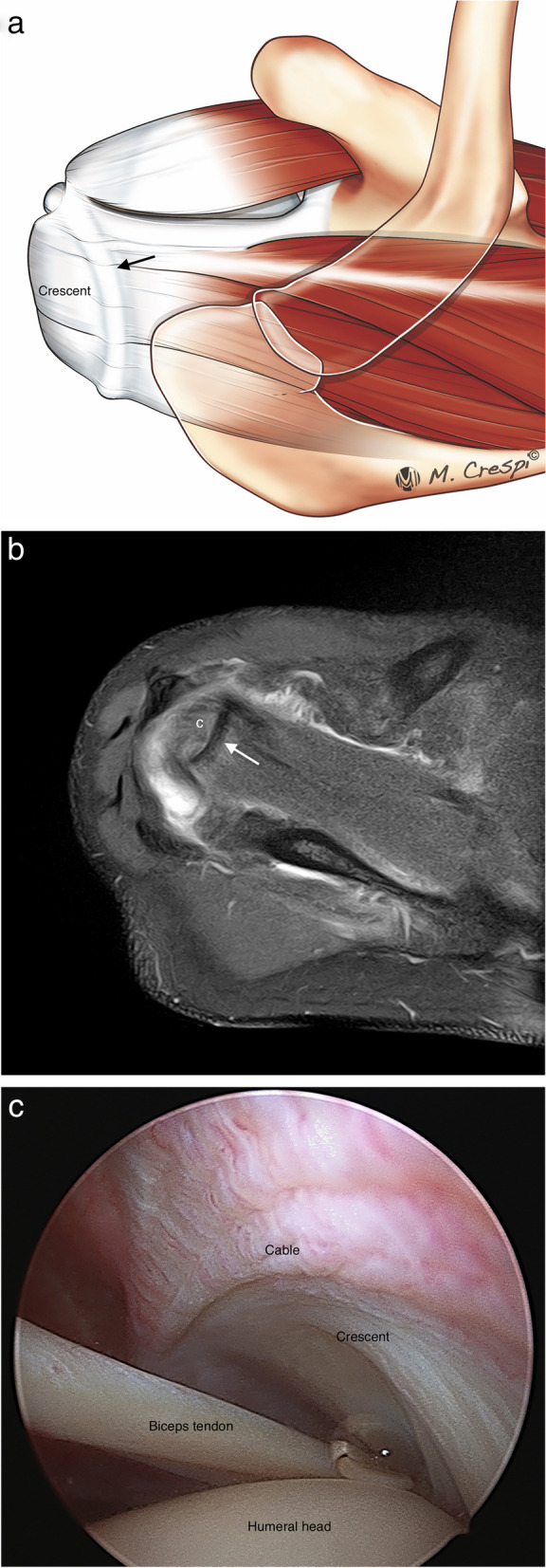
Normal rotator cable. **a** Drawing showing the cable and crescent. **b** Axial fat-suppressed PD-weighted MRI reveals the normal rotator cable (arrow) and the crescent (**c**) lateral to the cable. **b** Arthroscopic correlation view from the posterior portal showing the crescent and cable

Microscopically the fibers of the SS and IS are arranged in five layers, demonstrating the complex biomechanical role of the rotator cuff. Each layer has a different collagen fiber organization and orientation [7, 9]. Some of them can be identified with high-resolution MRI (Fig. 3). Layer 1 is the superficial layer of the coracohumeral ligament. Layer 2 is thicker (3–5 mm) with densely packed collagen fibers while layer 3 is less thick (3 mm) and formed by smaller bundles of collagen with a less uniform organization [8]. The fibers of layer 2 are more elastic, more resistant to rupture and are well vascularized. Conversely, the fibers of layer 3 are inelastic and more prone to tear and are poorly vascularized [8, 15]. The differences between layers 2 and 3 regarding the different movement between them, thickness, tensile properties, orientation of the collagen fibers, vascularization, and stress they support can explain the origin of partial and delaminating tears [4, 15, 16]. In the inferior part of the SS and IS, there is a bundle of connective tissue fibers perpendicular to the long axis of the SS and IS tendons, known as the rotator cable. The rotator cable is a capsular reinforcement formed by an extension from the deep layers of the coracohumeral ligament, this forms the 4th layer of the RC. Anteriorly the rotator cable has two insertions, on the superior facet of the lesser tuberosity and on the anterosuperior edge of the greater tuberosity. The posterior insertion is complex in the area between the middle and inferior facets of the greater tuberosity, between the insertion areas of the teres minor and the insertions of the SS and IS tendons [17]. The area lateral to the rotator cable is called the rotator crescent [17, 18]. The rotator cable acts as a suspension bridge, dissipating load from the rotator crescent which is particularly susceptible to tears, and inhibiting retraction of the SS or IS [18–20]. The rotator cable can be observed in most high-resolution MRI, especially with Arthro MRI [20] (Fig. 4). Some studies suggest that it is better observed on the ABER views, thanks to the relaxation of the rotator cuff in abduction and external rotation [21]. The last layer, the 5th, is the articular capsule [7].

**Fig. 5:**
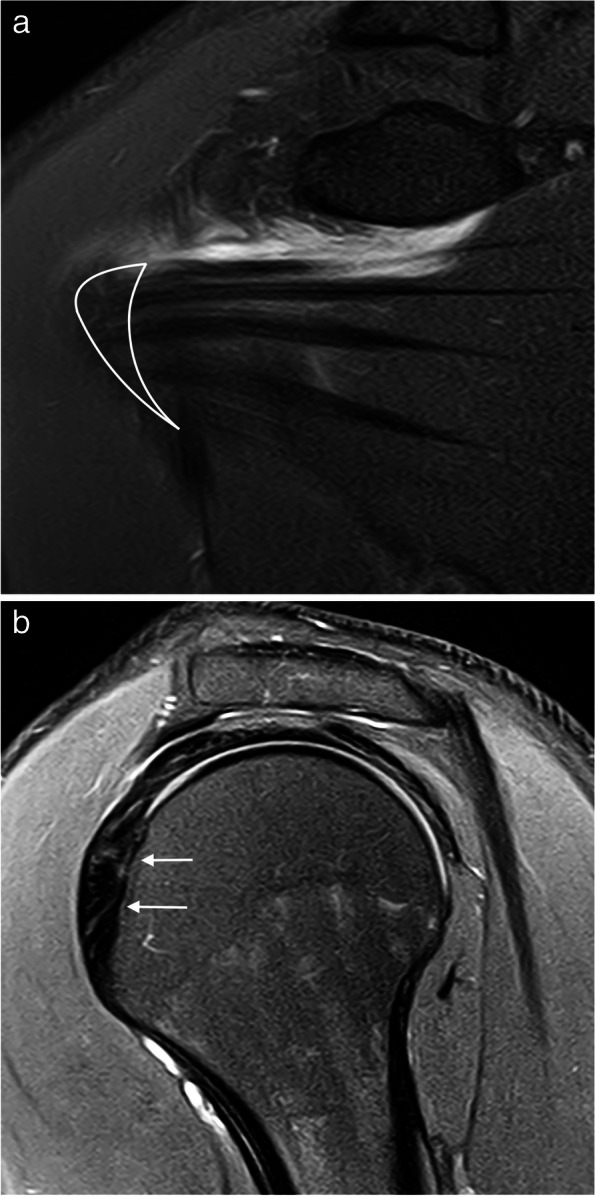
Normal subscapularis. **a** Oblique coronal fat-suppressed PD-weighted MRI showing the comma shape of the SSC insertion (white outline). **b** Oblique sagittal fat-suppressed PD-weighted FS MRI demonstrate the normal appearance of the tendon (arrows)

The subscapularis (SSC) is the largest and most powerful of the rotator cuff muscles [22]. It arises from the anterior scapula and has multiple tendinous bands that merge laterally to insert onto the lesser tuberosity of the humeral head in a broad comma-shaped footprint (Fig. 5). The two upper thirds of the insertion are tendinous and wider with variable attachment onto the lesser tuberosity, bicipital groove and greater tuberosity. The lower third has a muscular insertion on the inferior margin of the lesser tuberosity and in the anterior aspect of the humeral metaphysis [22, 23].

The two upper thirds are stronger, stiffer and subjected to greater tension while the inferior third is mechanically weaker [24, 25]. These factors may explain why some tears may be confined to either the superior or inferior portions of the SSC [22].

The upper fibers of SSC tendon interdigitate with the anterior fibers of SS tendon and the structures of the rotator interval. This anatomical disposition may explain why the SSC tendon tears are frequently associated with massive RC tears or rotator interval injuries [26].

## Rotator cuff tears

The location of RC tears can be divided according to ISAKOS [2] into posterosuperior, when they affect the supraspinatus (SS), the infraspinatus (IS) or unfrequently the teres minor (TM), and into anterior when they affect the subscapularis (SSC).

### Posterosuperior partial tears

Partial thickness RC tears are very common. There are some intrinsic and extrinsic factors involved in the pathogenesis of these tears. Depending on their location, they are divided into articular, bursal or interstitial [27].

**Fig. 6:**
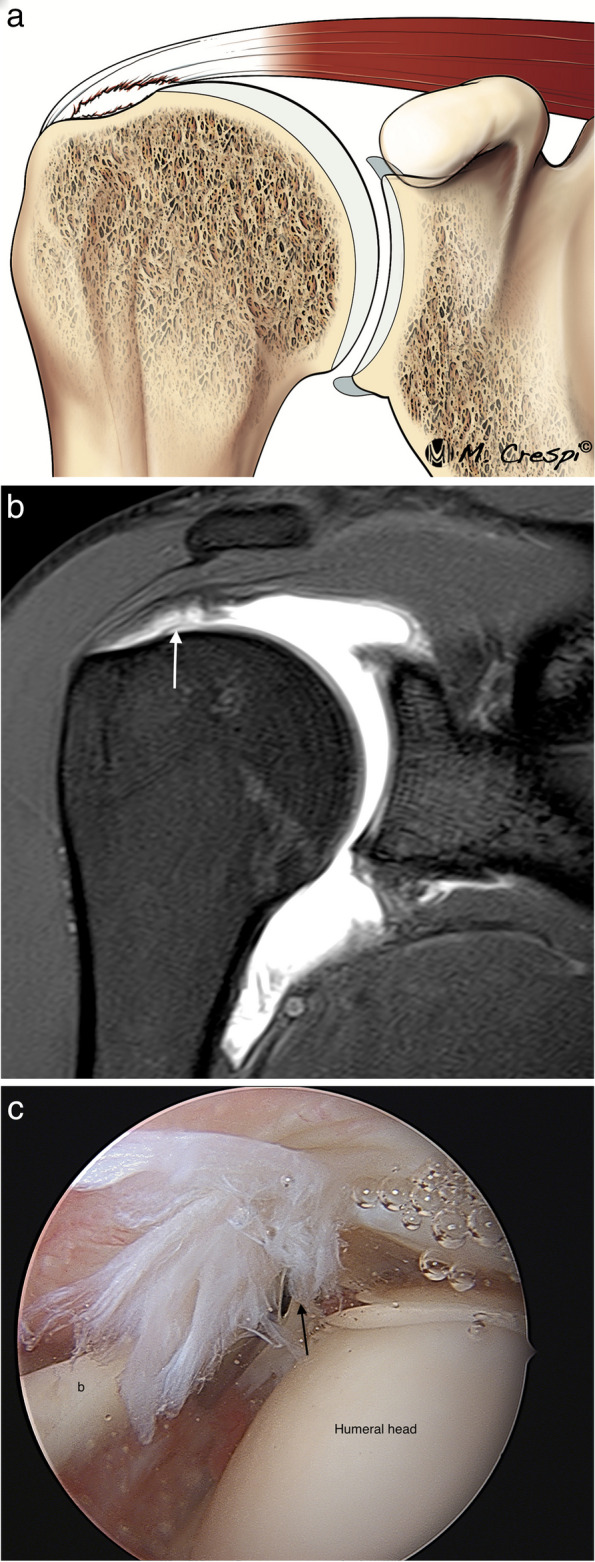
Partial articular side tear of the supraspinatus tendon. **a** Drawing showing the disruption of the articular layer of the SS. **b** Oblique coronal fat-suppressed PD-weighted MRI arthrography demonstrates a discontinuity and irregularity of the articular side of the tendon (arrow) affecting more than 50% of tendon thickness. **c** Arthroscopic view from the posterior portal confirming the tear with fraying of the articular side of the tendon (black arrow). *b* biceps tendon

Partial articular-side tears are the most frequent type of RC partial tear. They involve the fibers that are in contact with the humeral head (Fig. 6). These fibers are exposed to high tension, they are less elastic and more resistant to deformation making them more prone to tears. Their prevalence is not well known but is reported to range from 15 to 60% of all RC tears [15, 16, 27, 28]. Overhead athletes develop frequently posterosuperior impingement associating asymptomatic partial articular-sided rotator cuff with posterior superior labral tears. In athletes, these types of rotator cuff tears are managed differently [29]. Partial articular-sided tears have been named with different names such as RIM RENT or PASTA (partial articular supraspinatus tendon avulsion) [30, 31] but in our experience this nomenclature has no impact on the selection of treatment and is currently becoming less used.

**Fig. 7:**
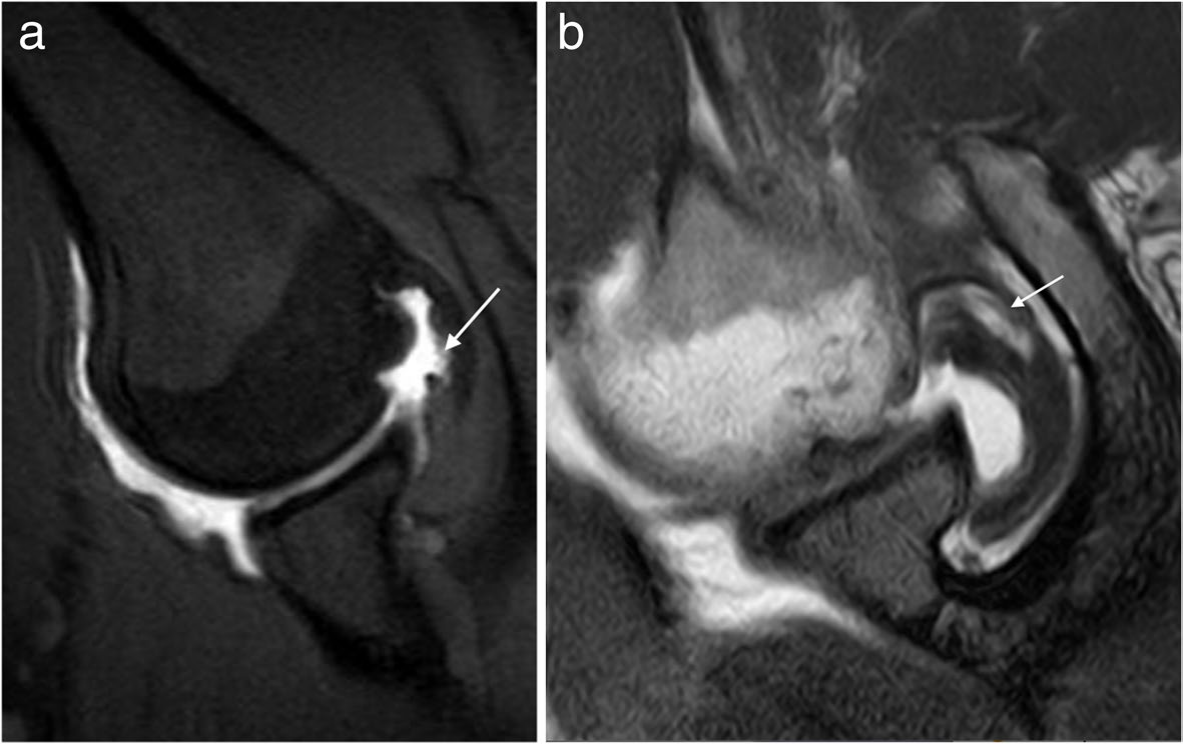
Partial articular-sided tear of the supraspinatus tendon (two different patients). **a** ABER fat-suppressed PD-weighted arthrographic MRI demonstrate the partial articular tear (arrow). **b** ABER T1-weighted MRI arthrography reveals an articular tear with horizontal intratendinous extension. Note how the contrast is filling the tear (arrow)

MR arthrography is more accurate than MRI in detecting partial articular surface RC tears. In addition, abducted and externally rotated position (ABER) improves its visualization [32, 33] (Fig. 7). The ABER position relaxes SSP fibers allowing contrast to enter the torn fibers and thus demonstrating better the horizontal extension of the tear, including its intratendinous extension (delamination). However, improvements on the resolution of MRI systems allow accurate diagnosis without needing intraarticular injections [34].

**Fig. 8:**
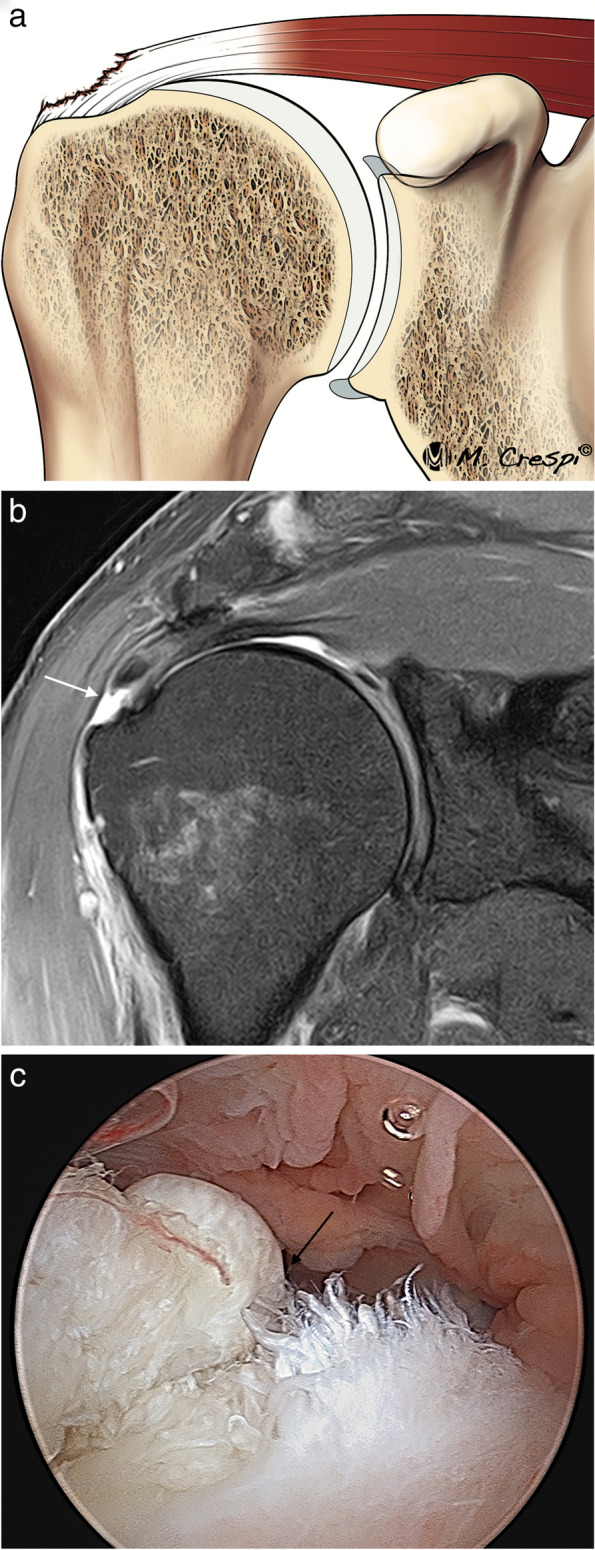
Partial bursal side tear of the supraspinatus tendon. **a** Illustration shows where the bursal side tears are located. **b** Oblique coronal fat-suppressed PD-weighted MRI reveals a discontinuity of the SS bursal side (arrow) greater than 50% of the tendon thickness. **c** Correlation with its arthroscopic view from a lateral portal where the tear is confirmed (black arrow)

Partial bursal-sided tears involve those fibers that are close to the subacromial bursa, they represent approximately 18% of partial tears [35] (Fig. 8). These fibers are less stiff than articular side fibers and spontaneous healing of these tears are unlikely to occur [36, 37].

Bursal-sided tears often are much more painful than even full thickness tears causing the patient to seek medical care more rapidly [38].

**Fig. 9:**
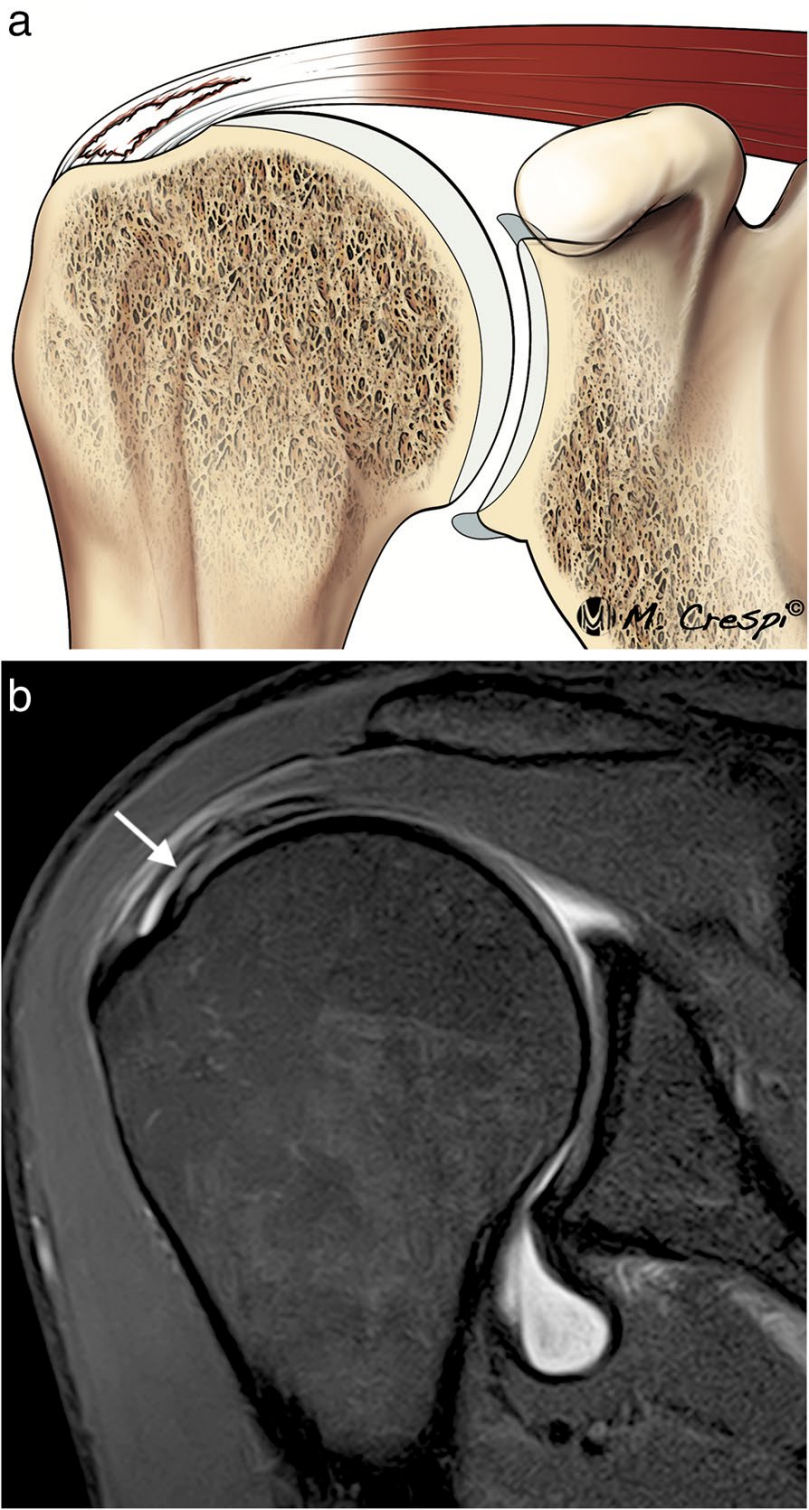
Partial intrasubstance tear of the supraspinatus tendon. **a** Illustration of the morphology of intrasubstance tears. **b** Oblique coronal fat-suppressed PD-weighted arthrographic MRI showing a tear contained within the thickening of the tendon (arrow)

Intrasubstance or intratendinous tears are located within the tendon substance and can be very painful. Some studies suggest that they are more frequent than articular tears but are often undiagnosed [15] (Fig. 9). These tears can be hidden at arthroscopy. MRI can demonstrate bursal and intratendinous tears with high sensitivity and specificity [39].

There have been different classification systems of RC partial tears. ISAKOS system tried to unify criteria and distinguishing between high-grade tears (i.e. those involving greater than 50% of tendon thickness) and low-grade tears (those involving less than 50% of tendon thickness) [2]. The rational is that high-grade tears have 55% chance to progress to full thickness while low-grade tears have only a 14% chance of progression [38, 40].

### Posterosuperior partial tear treatment

Treatment of posterosuperior partial thickness tears remains controversial, a major factor in making decisions is the percentage of the tendon thickness tear [2].

In the vast majority of cases, especially in low-grade tears, treatment initially is conservative and includes activity modification, avoidance of overhead or pain-provoking actions, NSAIDs and physical therapy. If conservative treatment fails, surgery is considered [16, 37, 40]. Bursal-sided partial thickness tears usually do not respond to conservative treatment; therefore, some authors recommend direct surgery [37, 38].

For high-grade partial thickness articular-sided RC tears, surgery can be considered as the first treatment option because of their tendency to progress to full thickness tear. Surgically treated high-grade tears have better outcome [2, 41, 42].

In overhead athletes, the treatment strategy for these high-grade articular-sided partial thickness tears is different. Surgery on high-grade tears leads to a decreased range of motion and stiffness; therefore, conservative treatment is recommended unless tears exceed 75% of the tendon thickness [18, 37].

There have been many different surgical techniques described, depending on the grade of the tear, on the devitalized tissue found during arthroscopy, but also on surgeons’ preferences. For low-grade partial thickness RC tears, treatment consists of arthroscopic debridement, with or without acromioplasty [28, 39]. For bursal-sided partial thickness tears of the RC, a novel and emerging technique is the application of a highly porous collagen biomatrix patch in order to reduce local shear and to increase tendon thickness [37]. For high-grade partial thickness tears, the recommended treatment is arthroscopic repair, of which various techniques have been described [16, 37, 40]. The two major arthroscopic repair techniques are as follows: the conversion repair technique in which a partial thickness tear is converted into a full thickness tear and thereafter the tear is repaired, and the in situ repair technique in which the goal is to preserve the existing anatomy. The most common in situ repair is the transtendon procedure where the anchors are placed through the tendon securing it to the bone. This surgery is usually performed for articular-sided tears [28]. For intrasubstance high-grade partial thickness tears, it is recommended to complete the tear and repair the tendon since the quality of the tendon tissue in this type of tear cannot be assessed during arthroscopy [43].

### Posterosuperior full thickness tears

**Fig. 10:**
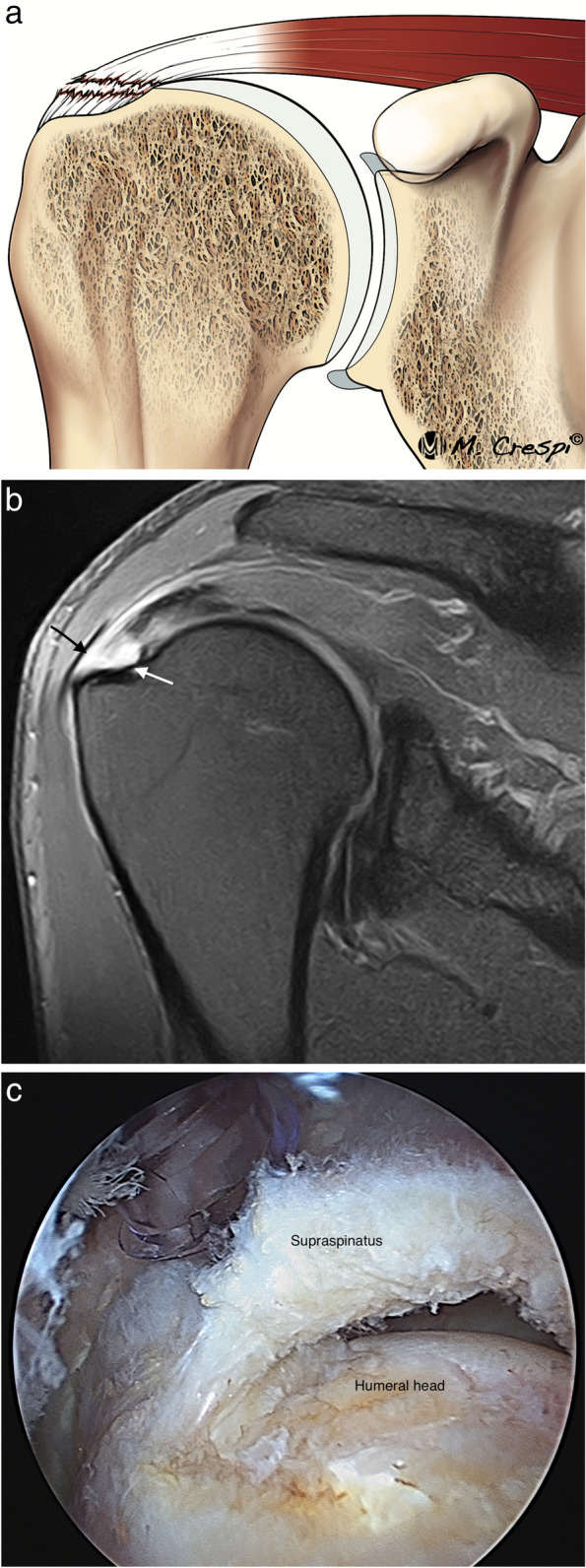
Full-thickness tear of the supraspinatus tendon. **a** Illustration of a full thickness tendon tear. **b** Oblique coronal fat-suppressed PD-weighted MRI showing a SS tendon tear that communicates the articular side (white arrow) and bursal side (black arrow). **c** Arthroscopic view from the lateral portal showing a complete tear and the communication between the articular and extra-articular spaces

A full thickness tendon tear is defined as a tear extending from the articular side to the bursal side of the tendon. It is important to understand that a full thickness tear does not imply the entire involvement of the anteroposterior diameter of the tendon (Fig. 10).

These types of tendinous lesions often involve the SS and IS. A teres minor full thickness tear is extremely rare [44].

Full thickness tears don’t have self-healing ability; without treatment, they progress, cause fatty atrophy of the muscle bellies, and can finally alter the normal shoulder kinematics leading to osteoarthritis [45].

**Fig. 11:**
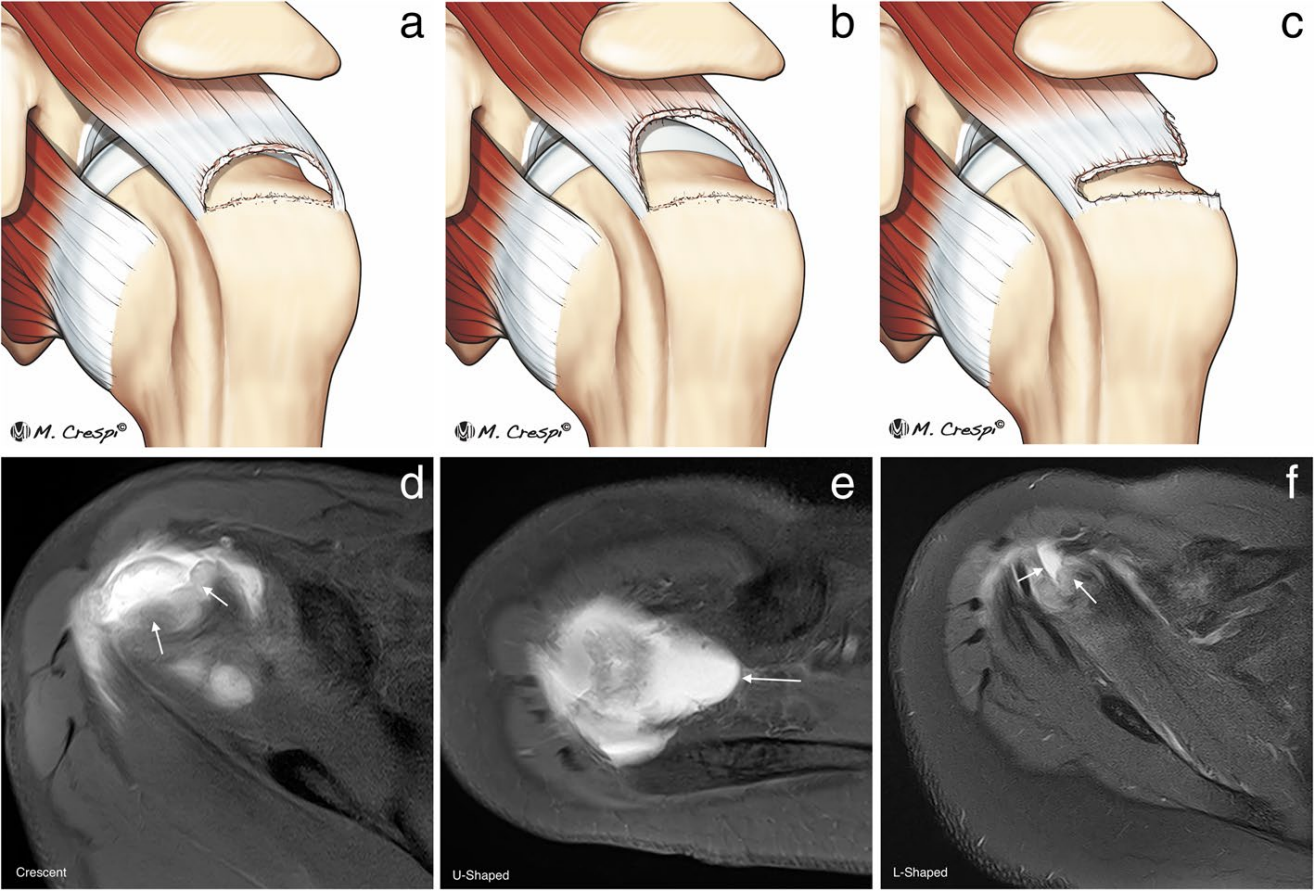
Full thickness tear patterns. **a**–**c** Illustrations of the different full thickness tear patterns: crescent-shaped, U-shaped, and L-shaped. **d**–**f** Axial fat-suppressed PD-weighted MRI arthrography showing the different patterns (arrows)

ISAKOS consensus recommends for full thickness tears MRI assessment of the pattern, the extension, and the retraction of the tendons [2]. The pattern description (four types) is based on the geometric shape assessed at arthroscopy as described by Davidson and Burkhart into crescent-shaped, U-shaped and L or inverted L shape [5] (Fig. 11). The recognition of these tear patterns is critical for anatomical repair during arthroscopy [5, 45]. Unfortunately, the correlation with MRI is difficult. Comparing the gap in coronal and sagittal images can help in assessing the morphology of the tear, and 3D MRI is a promising tool [46]. In the L-shaped tears, the gap is usually wider in oblique coronal images and shorter in sagittal oblique images while the crescent and U-shaped tears are wider in oblique-sagittal plane than in the oblique coronal images [2].

**Fig. 12:**
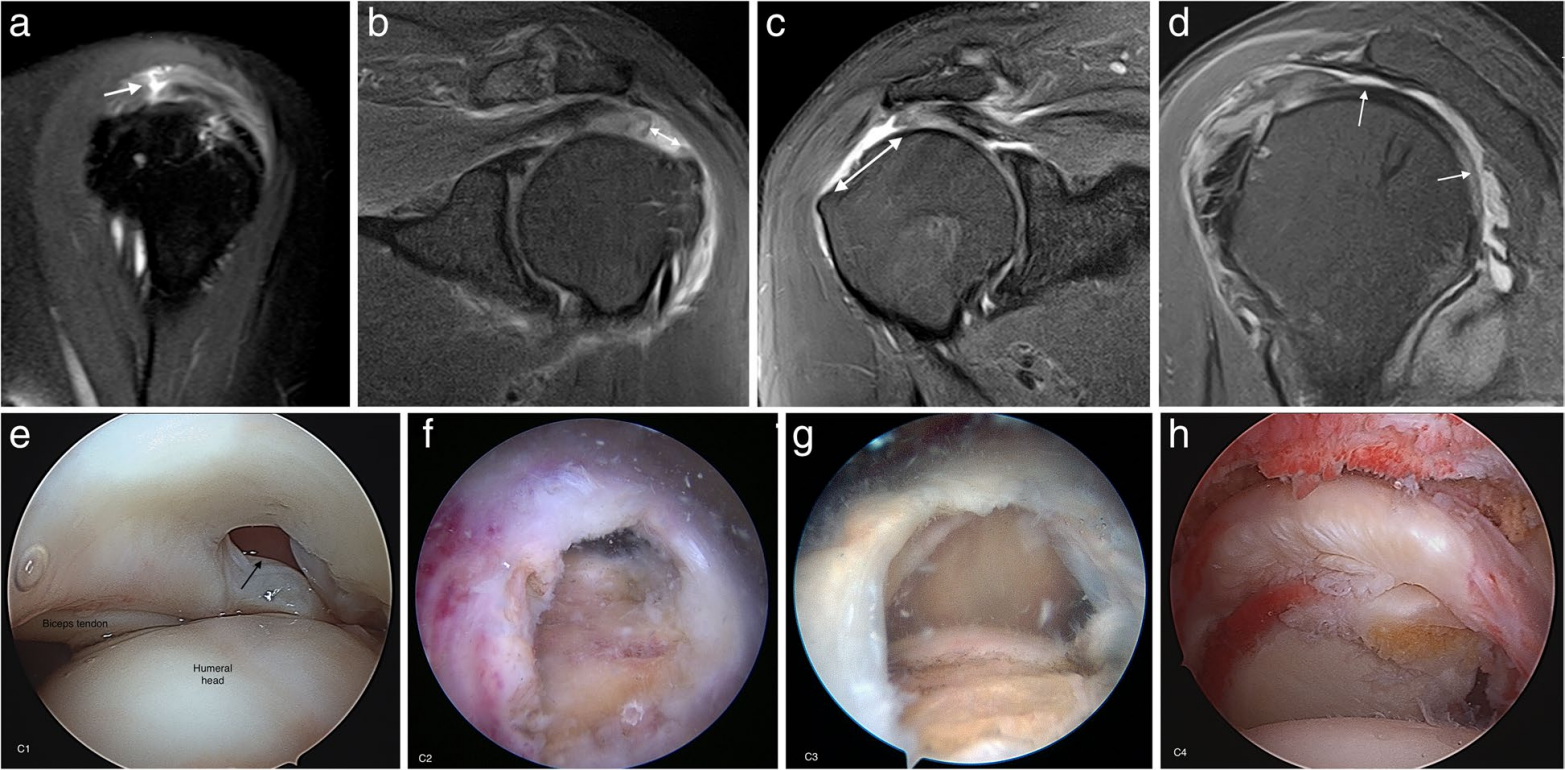
Full thickness tear extension. **a** Oblique sagittal fat-suppressed PD-weighted MR arthrography showing a C1 tear consisting in a small full thickness tear or pinhole (arrow). **b** Oblique coronal fat-suppressed PD-weighted MR demonstrates a C2 tear with a small retraction, less than 2 cm (double-headed arrow). **c** Oblique coronal and sagittal shows a C3 tear which is a large full thickness tear with a retraction usually between 3 and 4 cm (double-headed arrow) affecting only one tendon. **d** Oblique sagittal fat-suppressed PD-weighted MRI showing a C4 tear consisting in a complete tear of two or more tendons. In this case, there is a tear of both supraspinatus and infraspinatus (arrows). **e** Arthroscopic view of a C1 tear from the posterior portal (arrow). **f**–**h** Arthroscopic views of C2, C3, and C4 tears from a lateral portal

The extension of the tear is evaluated following the Snyder classification because it is the only system that provides information about the size of the tear in the coronal oblique plane, the number of tendons affected, and the degree of scarring [45]. C1 is a small full thickness tear with a pinhole; C2 is a moderate full thickness tear, smaller than 2 cm, involving one tendon without retraction; C3 is an extensive full thickness tear with retraction of 3 to 4 cm and C4 is a massive tear compromising two or more tendons and with marked retraction and scarring of the remaining tendon (Fig. 12). The tear extension is important for surgical planning; it can predict the mean surgical time and the release of soft tissues necessary for repair [2].

**Fig. 13:**
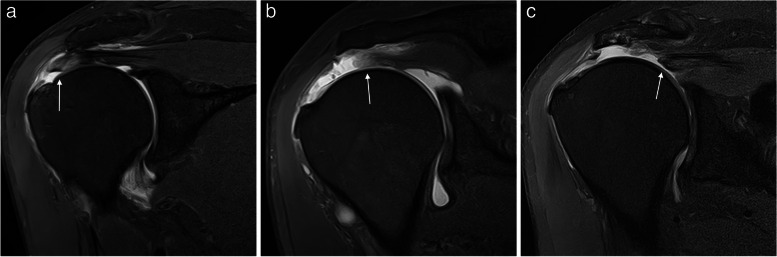
Supraspinatus full thickness tears different retraction stages. **a**, **b** Oblique coronal fat-suppressed PD-weighted MRI arthrography showing a complete full thickness tear with a small retraction (arrow in **a**) and a full thickness tear with fibers retracted over the humeral head vertex (arrow in **b**). **c** Oblique coronal fat-suppressed PD-weighted MRI shows tendon fibers retracted over the superior glenoid margin (arrow)

Retraction of the tendon has a prognostic value; the greater the retraction is the worse is the prognosis. The assessment of retraction was described by Patte as follows: grade I, tear with minimal retraction; grade II, fibers retracted over the vertex of the humeral head; and grade III, fibers retracted to the superior glenoid margin or more proximal (Fig. 13) [47].

### Posterosuperior tears variations

Other types of rotator cuff tears with special features that distinguish them from the classic classifications have been described. They are all very closely related and are variations of the main tears that we described above. However, these specifications might be significant for planning surgery or for establishing prognosis.

#### Delaminated tears

Delaminated tears are intratendinous horizontal tears splitting the articular and bursal layers of the SS and IS with or without retraction of the layers; they can be partial or full thickness tears [4]. The prevalence of these tears is variable among the different publications ranging from 38 to 82% of all RC tears [48, 49].

Anatomically, there are various factors predisposing to delaminating tears such as differences in elasticity, orientation of the fibers, and vascularization in layers 2 and 3 of the SS and IS [4, 48, 50]. Delaminated tears diminish the quality of the tendon tissue. The fluid separating the layers together with synovial hypertrophy adversely affects the healing potential. It represents a negative prognostic factor and requires a different surgical approach. Therefore, it is important to describe them on MRI to accurately plan surgery [51, 52].

**Fig. 14:**
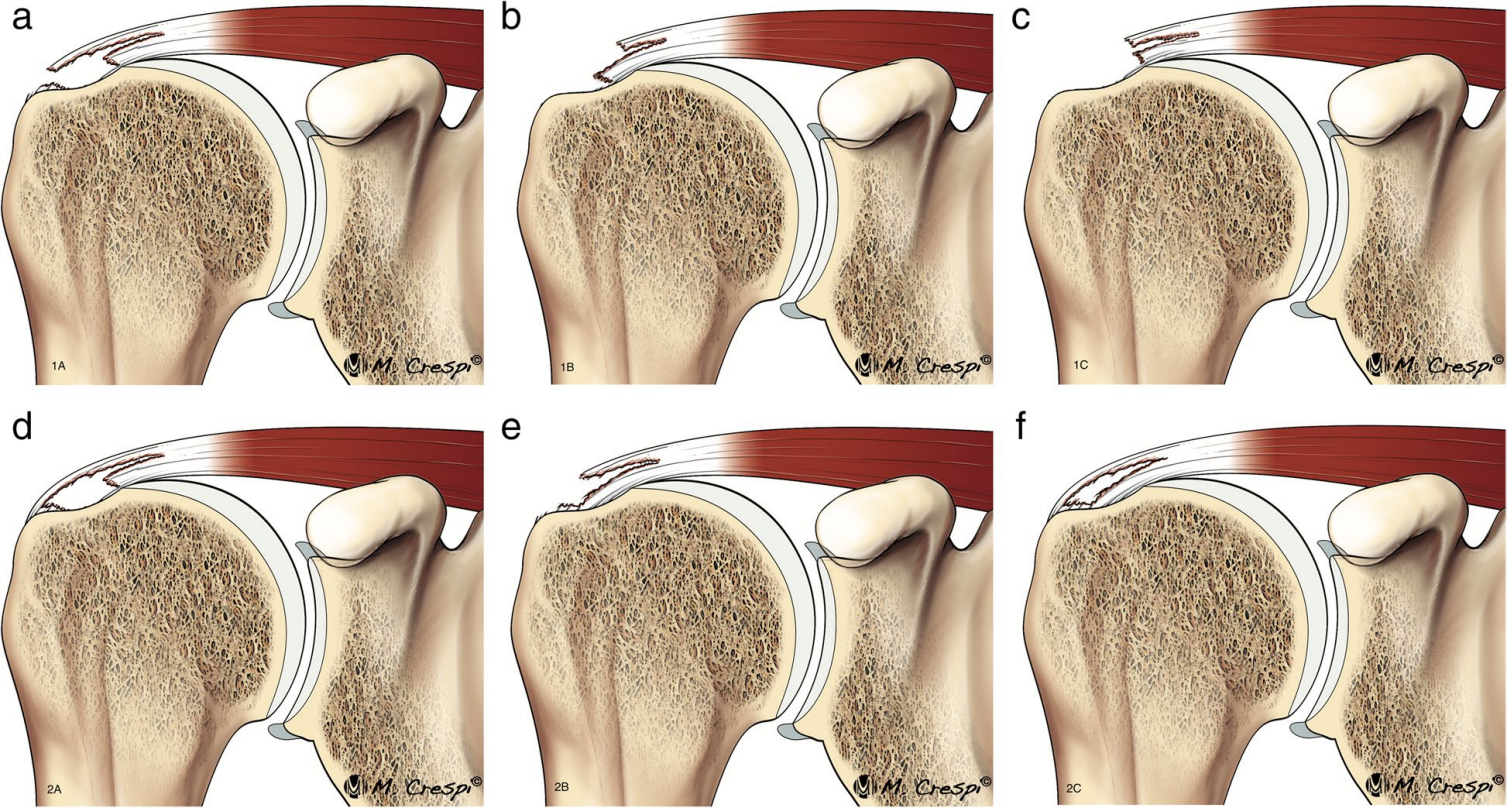
Delaminated tear classification. Illustrations showing the different types of delaminated tears. **a** Complete tear with articular layer more medially retracted than the superior layer. **b** Complete tear with bursal layer more medially retracted than the articular layer. **c** Complete tear with both layers equally retracted. **d** Partial thickness articular side tear with horizontal splitting extension. **e** Partial thickness bursal side tear with intratendinous splitting tear. **f** Intrasubstance tear with intratendinous extension

**Fig. 15:**
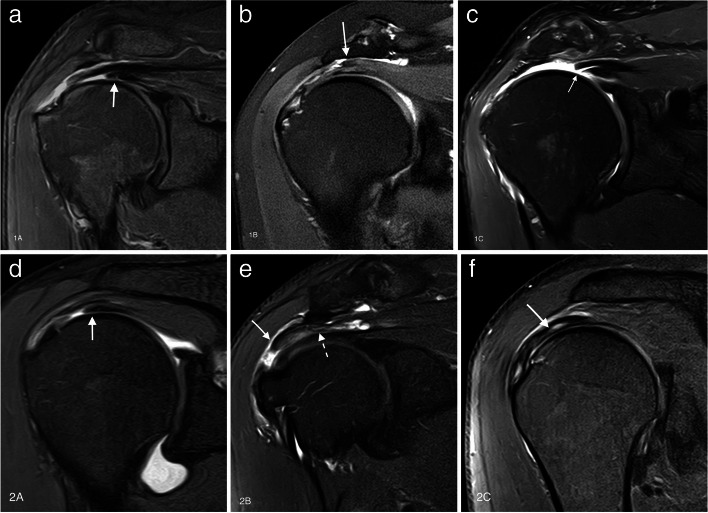
Delaminated tears of supraspinatus tendon. **a**–**f** Oblique coronal fat-suppressed PD-weighted MRI images. Full thickness delaminated tears. Articular layer more retracted than bursal layer (arrow in **a**). Bursal layer more retracted than articular layer (arrow in **b**) and bursal and articular layers equally retracted (arrow in **c**). Partial thickness delaminated tears. Articular side tear with intratendinous extension and retraction (arrow in **d**). Bursal side tear (arrow in **e**) with intratendinous extension (dashed arrow). Intrasubstance tear with horizontal intratendinous extension (arrow in **f**)

**Fig. 16:**
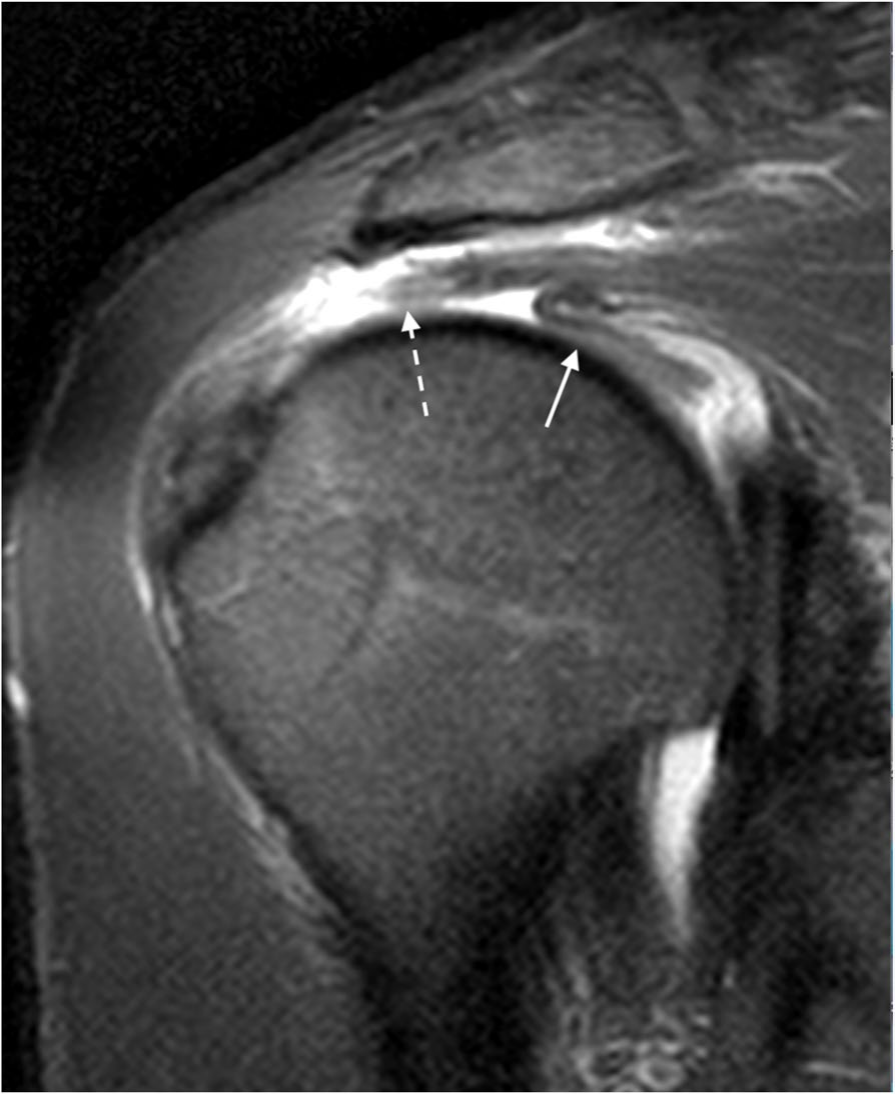
Full thickness delaminated tear of the supraspinatus tendon with articular layer flipped medially into the articular space. Oblique coronal fat-suppressed PD-weighted MRI reveals a complete SS tendon tear (dashed arrow) and note how the articular layer is flipped downwards into the articular space (arrow)

Delaminated tears are more common in the SS than in the IS [51]; however, both Mochizuki et al. and Choo et al. have found that the vast majority, up to 80%, of the delaminated tears of the SS occur in combination with those in the IS [4, 50]. This is secondary to the intertwined fibers of the SS and the IS. Choo et al. classified them into full thickness and partial thickness and subdivided them depending on the retraction of the articular or bursal layer [4] (Figs. 14, 15). The articular layer is more frequently retracted than the bursal layer. Exceptionally, the retracted articular layer can be flipped downwards (Fig. 16). Sometimes, it is difficult to demonstrate on MRI a partial thickness delaminated tear due to the absence of fluid between the layers.

Treatment of delaminated tears is complicated and differs from tears without delamination. Many techniques have been described but essentially the objective is to repair the layers separately in order to restore their original insertions and restore the function of the RC [52]. The deep layer (superior glenohumeral capsule, corresponding to layer 5 described above) is pulled laterally and fixed on the medial edge of the greater tuberosity and the superficial layer (SS or IS) is pulled anterolaterally and fixed on the lateral edge of the footprint on the greater tuberosity [50, 52].

#### Fosbury flop tear

**Fig. 17:**
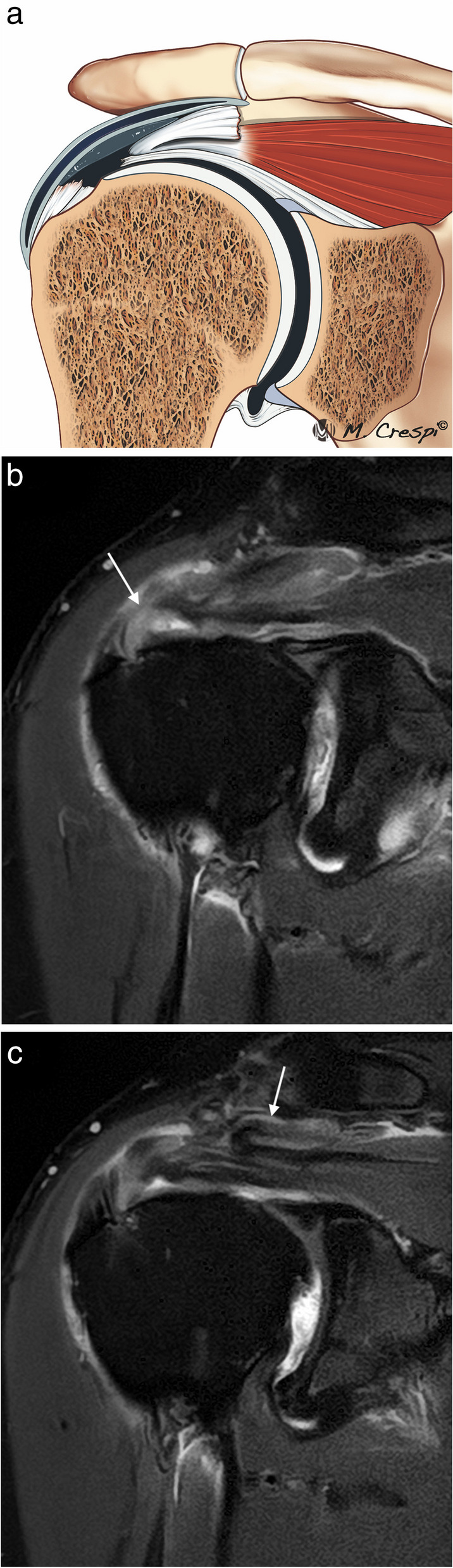
Flipped supraspinatus full-thickness tear “Fosbury flop tear”. **a** Draw illustrating the flipped SS tendon. **b**,** c** Oblique coronal fat-suppressed PD-weighted MRI demonstrate a full thickness tear of supraspinatus (arrow in **b**) with tendon stump flipped upon itself (arrow in **c**)

The concept of Fosbury flop tear was described by Ladermann et al. [53]. In a full thickness tear, the tendon stump can flip backwards upon itself, hence the name "Fosbury”, and adhere superomedial in the bursal-sided. On MRI, it can be recognized when the tendon stump is found to be thicker on the bursal-sided with a superomedial orientation (Fig. 17).

#### Intramuscular rotator cuff cysts

Intramuscular RC cysts are usually associated with underlying partial or full thickness RC tears. Intraarticular fluid enters the tear and dissects along the horizontal plane into the muscle, but secondary to the interdigitate structure of the RC fibers, they may propagate to the adjacent tendon or muscle. They are located within the sheath or intrasubstance of the rotator cuff tendons following the long axis of the fibers [54]. They might be occult at arthroscopy.

#### Myotendinous injuries

Tears of the myotendinous junction (MTJ) of the rotator cuff are uncommon but have a dramatic impact on functional outcome [55]. They can be observed in all muscles of the rotator cuff with a slight predominance in the IS [3, 56]. Myotendinous injuries of the SS usually involve the anterior bundle because of the greater contractile forces, and long intramuscular tendon components with bipennate configuration. Myotendinous junction lesions occur mainly in young patients (30 to 50 years old) and most of them are related to a recent history of trauma [57].

The tear starts in the MTJ, not in the tendon insertion, as a result from an indirect trauma mechanism consisting of forceful uncontrolled elongation during a phase of eccentric contraction brought on by muscle fatigue.

**Fig. 18:**
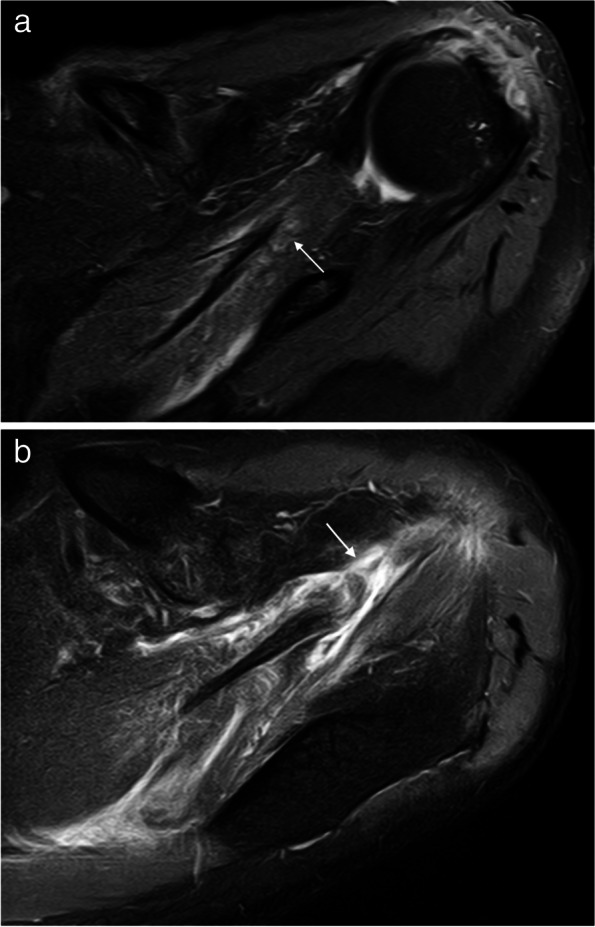
Supraspinatus myotendinous junction tears. **a**,** b** Axial fat-suppressed PD-weighted MRI. **a** Shows edema of the anterior muscle belly of the SS and a partial tear of the myotendinous junction (arrow). **b** Reveals a full thickness retracted tear of the myotendinous junction of the anterior belly of the SS (arrow)

They are classified into three stages [58]. Grade I: a muscular strain that heals without sequelae. MRI shows edema surrounding the MTJ but the fibrillar architecture of the muscle is preserved. Grade II: partial tears of the muscle fibers or the intramuscular tendon without tendon retraction. These are the most common. Edema or a focal hematoma is seen associated with a tear of the muscle fibers or the intramuscular tendon. Grade III: a complete tear of the MTJ with some muscle retraction causing loss of tension and leading to a rapidly progressive muscle atrophy with fatty infiltration (Fig. 18).

Most of the MTJs of the RC are treated conservatively. There is no consensus on its surgical treatment because repairing the MTJ does not prevent the atrophy of the muscle belly and there is not enough information on its clinical outcome [59, 60]. However, some authors prefer arthroscopic repair as early as possible in an attempt to prevent rapid progression of the fatty infiltration of the muscle belly [60].

#### Treatment of posterosuperior full thickness tears

Full thickness tears can be repaired arthroscopically or with open surgery (mini open).

Usually, they are repaired according to their pattern. In the crescent tears, the lateral free margin of the crescent tear can then be mobilized and fixed to the anatomic footprint without excessive tension [61], while the ¨U¨ or ¨L¨-shaped tears are usually repaired first, converging the margins to a crescent-shaped tear, and then fixed to the footprint [62]. The tendons can be fixed on the humeral head by a single or double-row technique. Most of the studies suggest that double-row fixation improves the mechanical properties of the repair [63] but overall single and double-row repairs are equivalent in terms of clinical outcome [64].

## Anterior tears

SSC tears are different from posterosuperior cuff tears, in their mechanism of injury and management. They are more difficult to recognize at both MRI and arthroscopy and therefore remain underestimated. Although the prevalence of SSC injuries is therefore not well known, it has been reported to be between 20 and 37% [65, 66].

Degenerative tears are the most frequent and begin in the upper third of the SSC and progress caudally and are associated in up to 25% of cases with posterosuperior RC tears [67].

**Fig. 19:**
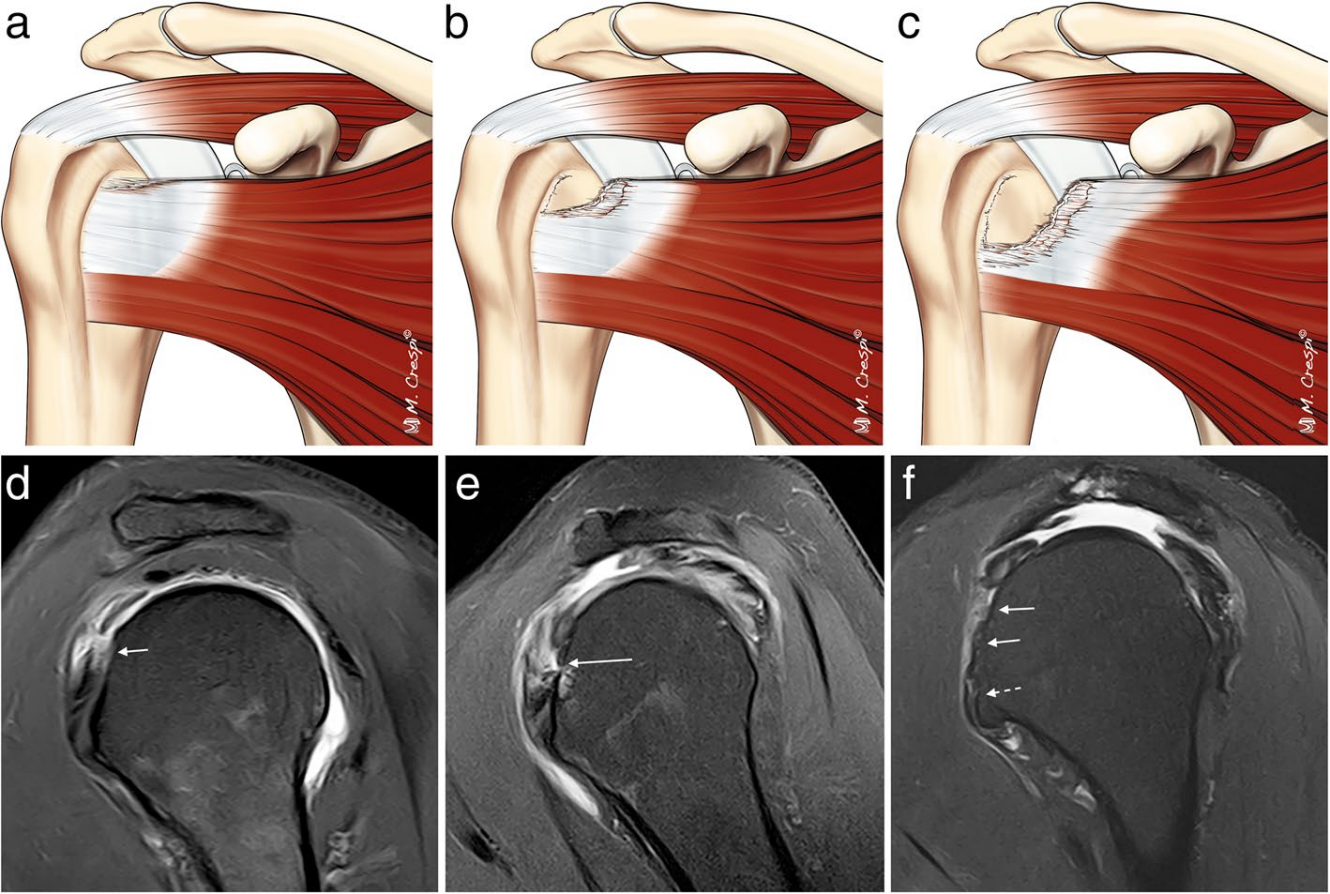
Anterior tears (subscapularis). **a**–**c** Illustrations of the different types (1 to 3) of the SSC tendon tears. **d**–**f** Oblique sagittal fat-suppressed PD-weighted MRI arthrography showing a partial tear of the upper third of the SSC tendon (arrow in **d**), a complete tear of the upper third of the tendon (arrow in **e**) and a complete tear of the upper and middle portions of the tendon (arrow in **f**) and intact lower third (dashed arrow)

**Fig. 20:**
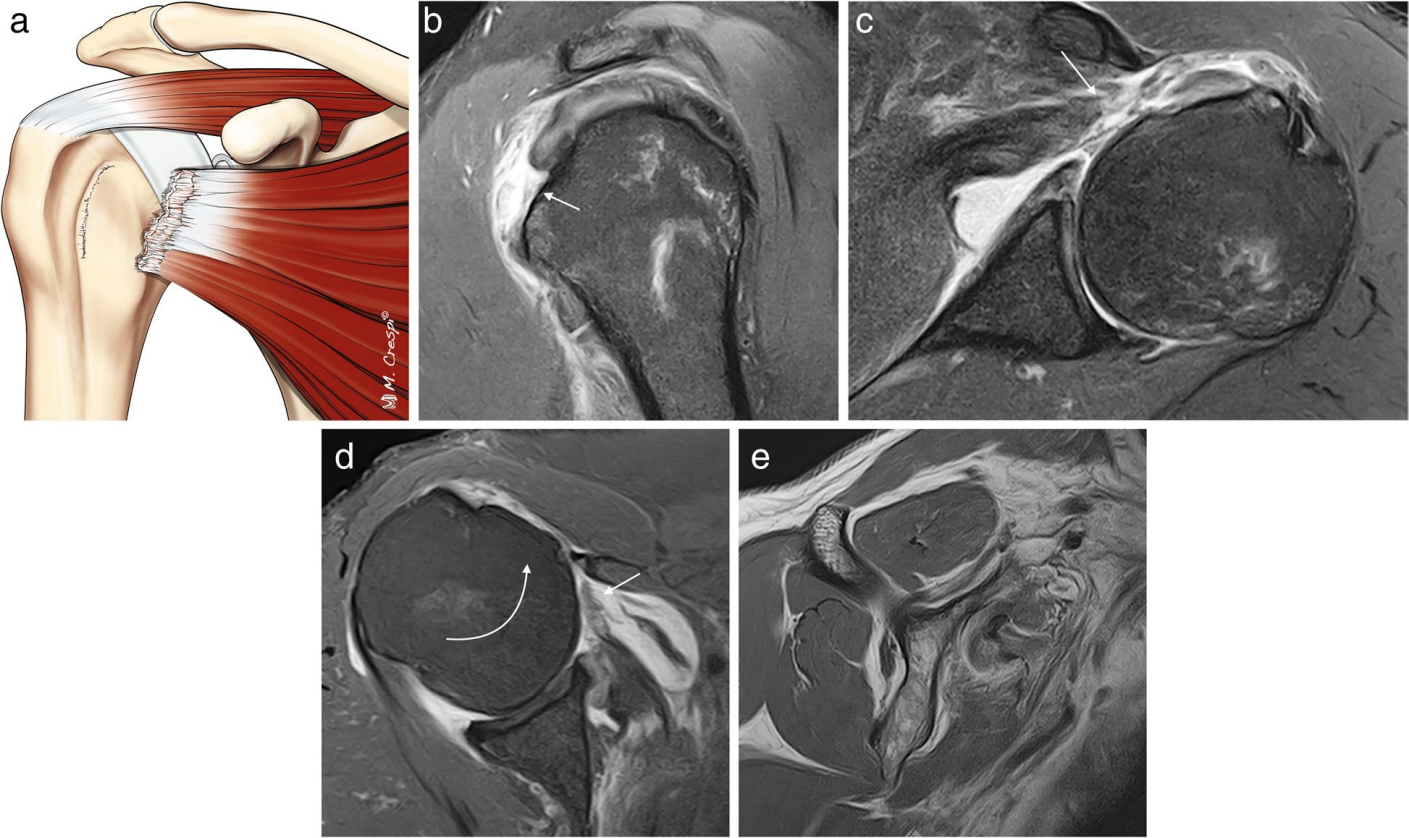
Anterior tears (subscapularis). **a** Illustration of type 4 and 5 tears of the SSC. **b**, **c** Axial and oblique sagittal fat-suppressed PD-weighted MRI showing a subscapularis full thickness tear (arrow) with retraction of the tendon (dashed arrow), note the humeral head is centered on the glenoid. **d**, **e** Axial fat-suppressed PD-weighted and oblique sagittal PD-weighted MRI images showing a type 5 tear (arrow) with anterior subluxation of the humeral head (curved arrow) and fatty infiltration Goutallier type 3

**Fig. 21:**
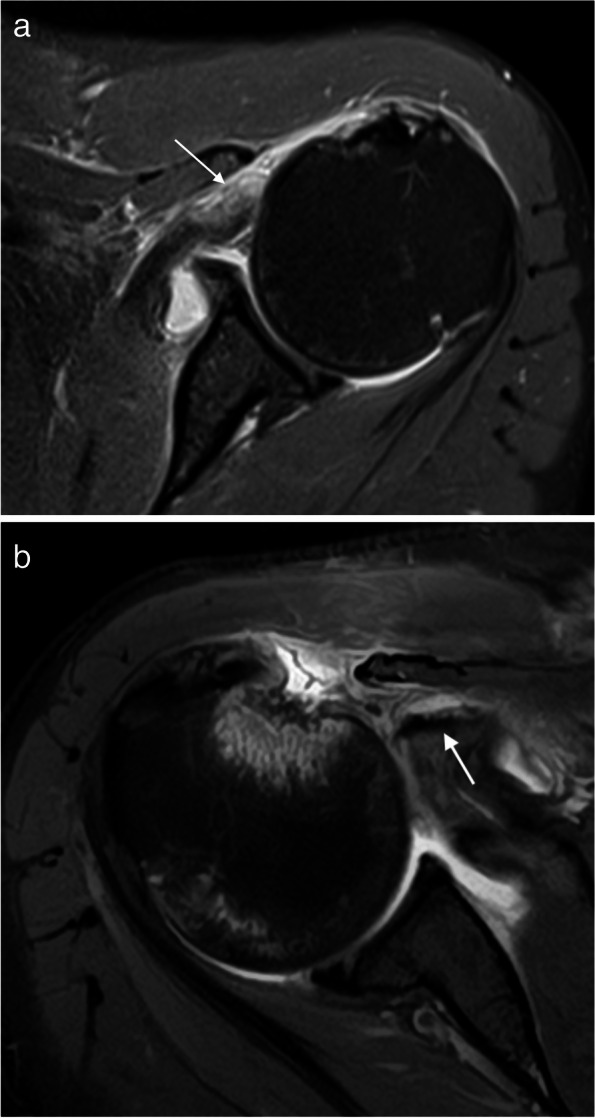
Traumatic subscapularis tears. **a**,** b** Axial fat-suppressed PD-weighted MRI images showing an acute SSC full thickness tear with retraction of the tendon (arrow in **a**) and an acute bony avulsion of the insertion of SSC tendon in the lesser tuberosity (arrow)

Despite there not being universally accepted classification system for degenerative SSC tears, the one proposed by Lafosse is often used and was endorsed by ISAKOS because it allows differentiation between reparable and non-repairable tears [68]. Type 1 is a partial thickness tear of the upper third at the articular surface only. Garavaglia subclassified this type in 1A when the tear is superficial or 1B when they involve the deeper fibers [66]. In type 2, there is full thickness involvement with complete detachment of the upper third. In type 3, the upper and middle third is involved. Type 4 is a full thickness tear of the tendon of the SSC while the humeral head remains centered in the glenoid fossa, and fatty atrophy is less *or equal* than Goutallier type 3. Type 5 is like a type 4 lesion, but with anterior subluxation of the humeral head, coracoid impingement and fatty infiltration is equal *or greater* than Goutallier type 3 (Figs. 19 and 20). The close relationship of the SSC muscle with the anterior fibers of the SS means that type 1 and 2 injuries are frequently associated with biceps injuries and tears of the anterior margin of the SS [66, 67], whereas types 3 and 4 are associated with extensive tears of the RC [68]. Isolated SSC tears are uncommon and are mainly secondary to a traumatic event with hyperextension or external rotation of the abducted arm and occur more frequently near the insertion site on lesser tuberosity [69, 70] (Fig. 21).

The diagnostic yield of MRI for the diagnosis of SSC tears seems to be less compared to the posterosuperior rotator cuff tears, especially for type 1 tears [66]. A combination of axial and sagittal planes together with higher resolution imaging have improved our accuracy. For subtle tears, a small amount of fluid between the lesser tuberosity and the SSC tendon is a useful radiological sign. MR arthrography has a higher sensitivity and specificity for the diagnosis of these types of injuries [71]. In our experience, adding a sequence in the axial plane with external rotation of the shoulder during MR arthrography improves the visualization of the SSC attachment and outlines better the extent of subtle tears.

Large tears of the SSC might involve the anterior fibers of the SS and part of the rotator interval.

In patients with RC tears, a band-like structure can be observed connecting the cranial portion of SSC and the anterior margin of SS through the subcoracoid and subacromial space. This attachment of both tendons creates the appearance of an anterior bridge (“bridging sign”) or comma shape corresponding to a combined tear of the SSC tendon, anterior portion of SS, coracohumeral ligament, and superior glenohumeral ligament adhered to the anterior margin of SS tendon on arthroscopy [26, 72].

**Fig. 22:**
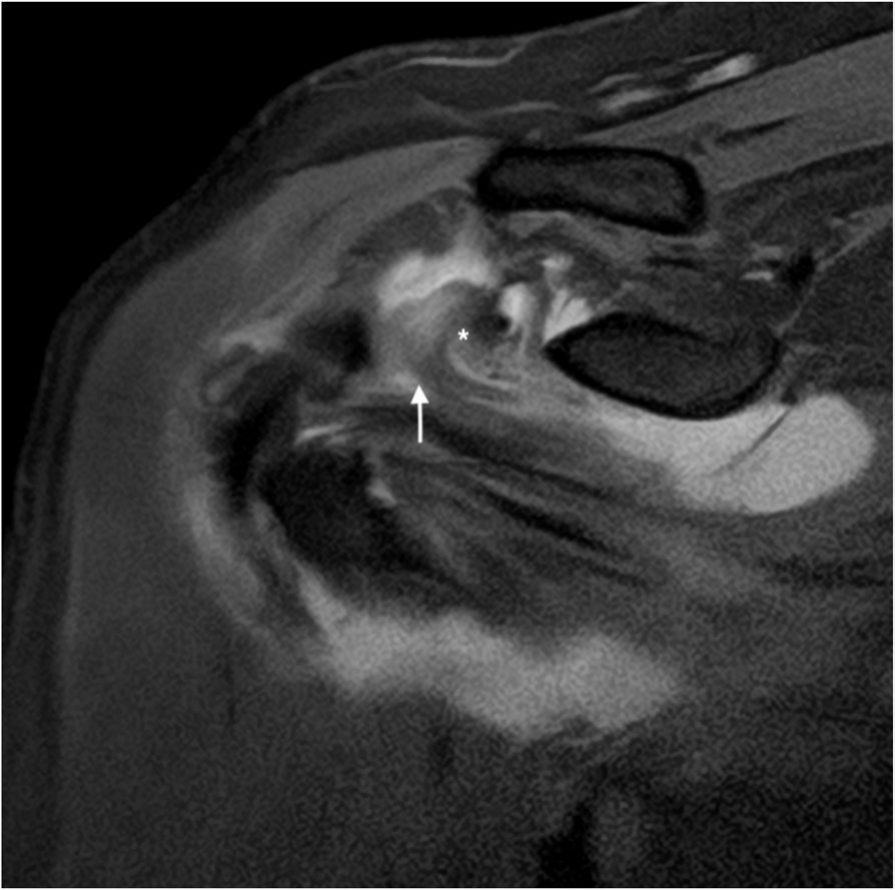
Bridging sign. Oblique coronal fat-suppressed PD-weighted MRI showing the torn cranial portion of the SSC tendon with superomedial displacement (arrow) due to retraction of the coracohumeral and superior glenohumeral ligament complex (asterisk)

Also, this sign is associated with more chronic shoulder pain and more severe rotator cuff tears [26] (Fig. 22).

### Anterior tear treatment

Most SSC tears can be repaired arthroscopically. Degenerative type 1 tears can be treated conservatively [72, 73]. For those patients that do not improve after conservative treatment, surgery is recommended; for type 1A, surgical debridement can be performed whereas for type 1B repair is usually necessary [67].

The treatment for grade II and III tears is to repair and fix of fixation to bone with one or two anchors as needed. For grade IV and V injuries, repair is also done with anchors using a single or double row [73, 74].

## Fatty infiltration

Chronic tears of the rotator cuff with involvement of the myotendinous unit lead to retraction with subsequent muscle atrophy and fibrosis of the fibers and fatty infiltration in the interfascicular and intrafascicular structure of the muscle. This phenomenon is progressive and irreversible. Deposit of lipids in muscle causes loss of elasticity and diminishes its compliance, making it more difficult to mobilize and repair RC components and increasing the chances of surgical failure [75]. The role of the suprascapular nerve has also been proposed as a factor that increases fatty infiltration of rotator cuff muscles. Retraction of the tendon and muscle induces excessive tension to the nerve, decreasing the propagation of neuromuscular junction potentials and reducing muscle contraction causing nerve stretching and, if chronic, denervation. This suprascapular nerve factor might explain the frequently seen fatty atrophy of the infraspinatus in supraspinatus tears [76].

Fatty infiltration of the rotator cuff is a major prognostic factor for the structural and functional outcome of rotator cuff surgery and is associated with a higher rate of retear [75, 76]. After repair, atrophy of the muscle belly may improve but the degree of fatty infiltration does not change [77].

**Fig. 23:**
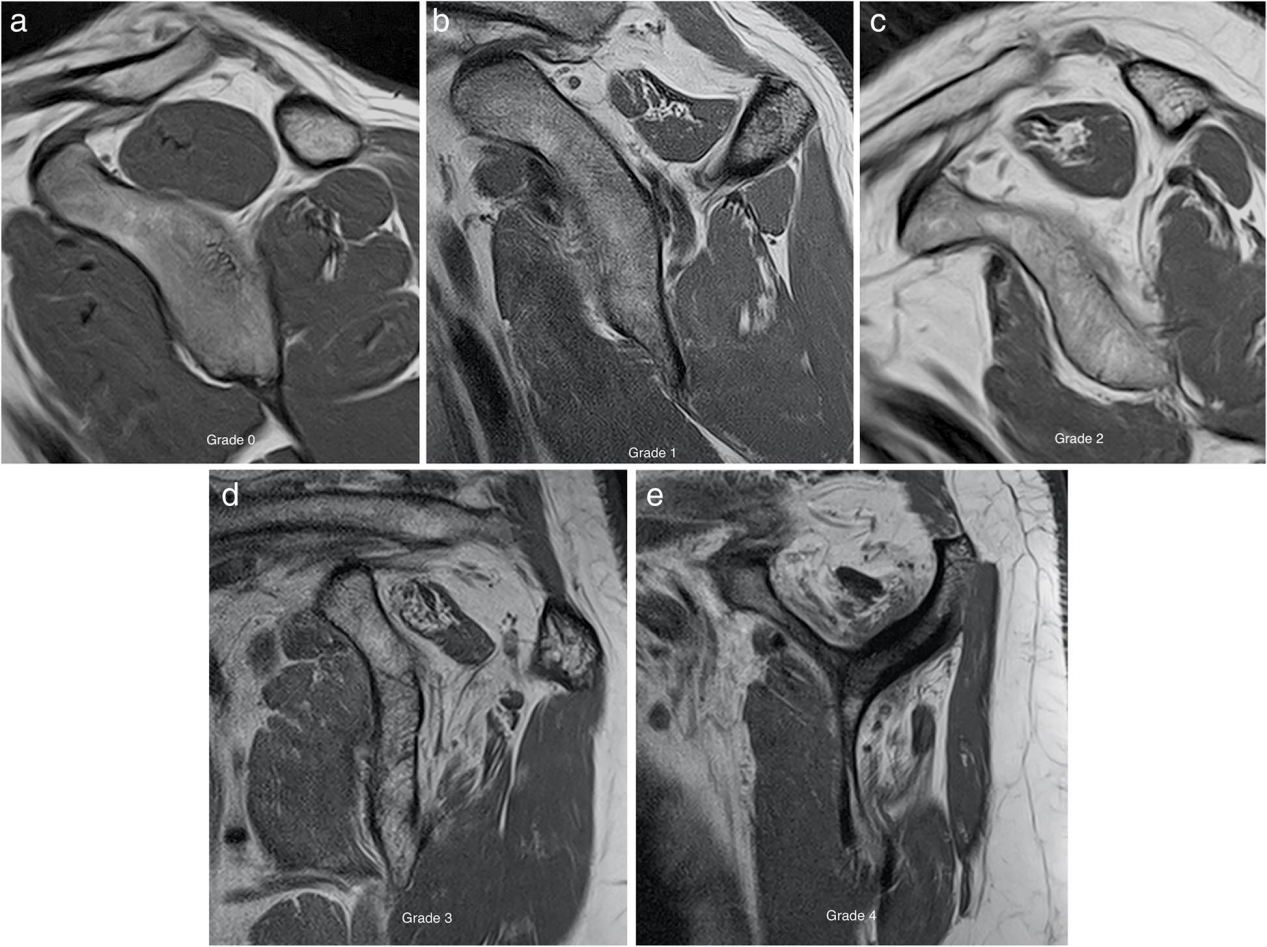
Fatty infiltration. **a**–**e** Oblique sagittal T1-weighted MRI showing the different grades of fatty atrophy following Goutallier modify classification for SS and IS tendons. Grade 0 = normal muscle, grade 1 = some fatty streaks, grade 2 = less than 50% fatty muscle atrophy (more muscle than fat), grade 3 = 50% fatty muscle atrophy, (equal muscle and fat), and grade 4 = more than 50% muscle atrophy (more fat than muscle)

It is important to assess the degree of fatty infiltration of the different RC muscles. Different systems have been described. Goutallier created a classification system with five stages [78] for assessing fatty infiltration of supraspinatus muscle based on CT (Grade 0 = normal muscle, Grade 1 = some fatty streaks; Grade 2 = less than 50% fatty muscle atrophy, i.e., more muscle than fat; Grade 3 = 50% fatty muscle atrophy, i.e., equal muscle and fat; Grade 4 = more than 50% muscle atrophy, i.e., more fat than muscle) (Fig. 23). Later, Fusch et al. validated this system to be used in MRI. Fatty atrophy is evaluated on the sagittal plane at the level of the coracoid process on T1-weighted images [79]. Other classification systems based on MRI have been used, such as the tangent sign described by Thomazeau. This is an easy method that categorizes volume loss of the SS on the sagittal plane as minimal, moderate, or severe with the disadvantage that it only considers the upper middle of the SS [80].

It is important to asses terer minor integrity and its fatty atrophy. The functional importance of teres minor is critical, especially in IS full thickness tear, since it becomes the only external rotator of the shoulder. A tear or severe fatty atrophy of the teres minor can affect postoperative clinical outcomes in patients with rotator cuff pathology and with latissimus dorsi tendons transfers [44, 81].

## Massive tears and rotator cuff arthropathy

The definition of massive tears is still under controversy. Initially, De Orio and Coefield defined massive RC tears as those tears being equal or greater than 5 cm in the coronal oblique plane [82]. Later, Gerber et al. considered massive rotator cuff tears when there were full thickness tears of two or more tendons [83]. Burkhart defined them based on shape and mobility of the tear and considered anatomical and functional factors that cannot be assessed by imaging [84]. Gerber’s definition is more consistent and has implications on functional and surgical outcome and is preferred by the surgeons [2].

Massive RC tears, which are more frequent in posterosuperior rotator cuff tendons [85] result in structural changes in the myotendinous unit with loss of elasticity, scarring, adhesion, retraction, fatty infiltration, and hypovascularity [86, 87]. Significant loss of mechanical and cohesive stability of the glenohumeral joint ensues. The force imbalance produced by massive tears generates superior migration of the humeral head. This migration is enhanced by the superior force exerted by the deltoid muscle as the posterosuperior RC do not longer act as a hinge and inferior force vectors, causing a clinical condition known as pseudoparalysis [88]. This severe impact of massive RC tears on shoulder biomechanics leads to a complex pattern of progressive secondary osteoarthritis [88, 89]. Therefore, early treatment is indicated.

The “term RC tear arthropathy” is also variable but the three major features are as follows: RC massive tear, glenohumeral degenerative changes, and superior migration of humeral head [85]. It is still unknown why some patients with RC tears progress to RC arthropathy and others do not. Several theories from crystal deposition induced arthropathy to mechanical factors have been proposed [87].

**Fig. 24:**
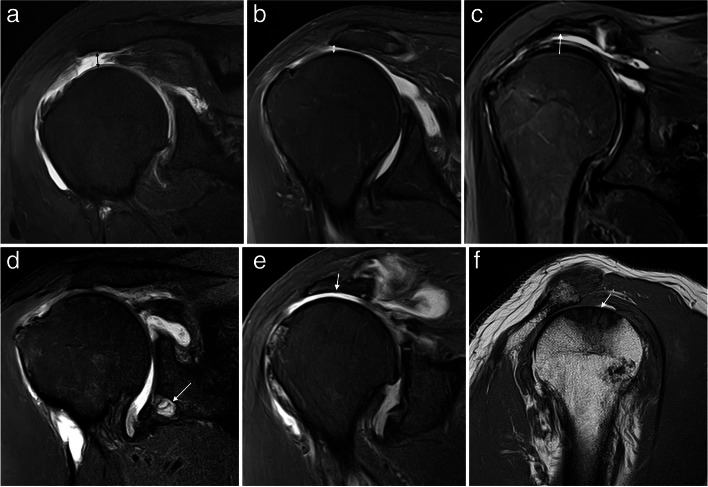
Hamada rotator cuff arthropathy evolutive stages. **a**–**c** Oblique coronal fat-suppressed PD-weighted MRI. Stage 1 showing an acromiohumeral interval (AHI) > 5 mm (double-headed arrow in **a**). Stage 2 the AHI is < 5 mm (double-headed arrow in **b**). Stage 3 is same as stage 2 but with the acetabularization of the acromion undersurface (arrow in **c**). **d**, **e** Oblique coronal fat-suppressed PD-weighted MRI. Stage 4a is similar to stage 2 but with degenerative changes in the glenohumeral joint (arrow in **d**). Stage 4B adds acromion acetabularization (arrow in **e**). **f** Sagittal oblique T1 MRI. Stage 5, there are osteonecrotic changes in the humeral head vertex (arrow in **f**)

Hamada et al. described in 1990 the radiological findings of rotator cuff arthropathty and classified it into five consecutive stages [90]. Although they were described on plain films, MRI is reliable for diagnosing and staging rotator cuff arthropathy [89] (Fig. 24).

They suggested that massive cuff tears will progress to RC arthropathy through a set of pathomechanics. Initially, the deltoid contraction during elevation causes superior migration of humeral head decreasing the acromiohumeral interval. The mechanical friction of the humeral head and the acromion and the increasing stress into the long head of the biceps tendon leads to a tear of the tendon and this subsequently contribute to the narrowing of acromiohumeral interval. If the superior migration continues, the humeral heads abuts the acromion leading to acromial acetabularization, femoralization of the humeral head, chondral damage, bone loss, and glenoid remodelation and retroversion.

### Massive tear treatment

Although massive tears are a therapeutic challenge, treatment including repair is often feasible. Many patients with massive tears of the rotator cuff have a good range of motion and can perform most of the daily activities. These patients are initially treated conservatively [91]. When conservative treatment fails, surgery, based on damage to anatomical structures, is considered. For reparable massive tears, complete anatomic repair should be performed, if possible, following the same surgical approach of full thickness tears. However, for irreparable tears, depending on the age and functional demand of the patient, the surgical procedure will be different.

In patients with high functional demand and mild arthropathy (Hamada I to III), it is possible to perform salvage surgery in order to delay the placement of a prosthesis. For those patients with low functional demand and moderate to severe arthropathy (Hamada IV/V), arthroplasty is the preferred technique [88, 91, 92].

## Salvage surgeries

In the last few years, several salvage surgeries, including augmentation techniques, have been advocated and used for the treatment of irreparable massive tears, especially in patients with high functional demand.

### Patch augmentation

Many patches for augmentation are available (non-degradable structures, extracellular matrix–based patches, and degradable synthetic scaffolds) [93].

### Superior capsular reconstruction

Superior capsular reconstruction restores superior shoulder stability and improves shoulder function and consists of placing a patch graft (biologic or synthetic) which is fixed with anchors in the upper glenoid margin and in the greater tuberosity [94, 95].

### Tendon transfers

Tendon transfers of latissimus dorsi, or lower trapezius for posterosuperior massive tears, are also an alternative for massive repairs but are technically demanding [96, 97].

### Subacromial spacer

Subacromial spacer consists of inflation of a reabsorbable balloon spacer, with saline solution, in the subacromial space for 3 months. It still is an evolving procedure [98, 99].

## Conclusion

Rotator cuff tears are a common cause of pain and disability, being variable in location, tear pattern, functional impairment, and reparability.

Radiological findings of the RC tears have a decision-making impact for patients, depending on tear pattern, extension, retraction and fatty atrophy.

 We recommend following ISAKOS consensus guidelines as they use radiological features in the decision-making process for partial, complete, and massive rotator tears. However, some specific patterns of tears, such as delaminated or myotendinous, do not fit in this classification and should be reported in addition.

 MRI can depict almost all of the various features of rotator cuff tears.

Radiologists should be familiar with the different surgical techniques and their decision-making factors to be able to deliver a useful radiological report and to help manage the patient.

## Data Availability

Not applicable.

## Abbreviations

ABER: Abduction and external rotation
AHI: Acromiohumeral interval
IS: Infraspinatus
ISAKOS: International Society of Arthroscopy, knee surgery & orthopedic sports medicine
MRI: Magnetic resonance imaging
MTJ: Myotendinous junction
PASTA: Partial Articular-Side Supraspinatus Avulsion
RC: Rotator cuff
SS: Supraspinatus
SSC: Subscapularis
